# Antiplatelet and antithrombotic effects of cordycepin-enriched WIB-801CE from *Cordyceps militaris* ex vivo, in vivo, and in vitro

**DOI:** 10.1186/s12906-016-1463-8

**Published:** 2016-12-07

**Authors:** Hyuk-Woo Kwon, Jung-Hae Shin, Deok Hwi Lim, Woo Jeong Ok, Gi Suk Nam, Min Ji Kim, Ho-Kyun Kwon, Jun-Hee Noh, Je-Young Lee, Hyun-Hong Kim, Jong-Lae Kim, Hwa-Jin Park

**Affiliations:** 1 Department of Biomedical Laboratory Science, College of Biomedical Science and Engineering, Inje University, 197, Inje-ro, Gyungnam, Gimhae, 50834 Korea; 2 Central Research Center, Whanin Pharm. Co., Ltd., 107, Gwanggyo-ro, Suwon, Gyeonggi-do 16229 Korea

**Keywords:** WIB-801CE, Cordycepin, Platelet aggregation, TXA_2_, Serotonin, Thromboxane A_2_ synthase, Arachidonic acid release, p^38^^MAPK^, ERK2, Thrombus

## Abstract

**Background:**

A species of the fungal genus *Cordyceps* has been used as a complementary and alternative medicine of traditional Chinese medicine, and its major component cordycepin and cordycepin-enriched WIB-801CE are known to have antiplatelet effects in vitro. However, it is unknown whether they have also endogenous antiplatelet and antithrombotic effects. In this study, to resolve these doubts, we prepared cordycepin-enriched WIB-801CE, an ethanol extract from *Cordyceps militaris*-hypha, then evaluated its ex vivo, in vivo, and in vitro antiplatelet and antithrombotic effects.

**Methods:**

Ex vivo effects of WIB-801CE on collagen- and ADP-induced platelet aggregation, serotonin release, thromboxane A_2_ (TXA_2_) production and its associated activities of enzymes [cyclooxygenase-1 (COX-1), TXA_2_ synthase (TXAS)], arachidonic acid (AA) release and its associated phosphorylation of phospholipase C_β3_, phospholipase C_γ2_ or cytosolic phospholipase A_2_, mitogen-activated protein kinase (MAPK) [p^38 MAPK^, extracellular signal-regulated kinase (ERK)], and blood coagulation time in rats were investigated. In vivo effects of WIB-801CE on collagen plus epinephrine-induced acute pulmonary thromboembolism, and tail bleeding time in mice were also inquired. In vitro effects of WIB-801CE on cytotoxicity, and fibrin clot retraction in human platelets, and nitric oxide (NO) production in RAW264.7 cells or free radical scavenging activity were studied.

**Results:**

Cordycepin-enriched WIB-801CE inhibited ex vivo platelet aggregation, TXA_2_ production, AA release, TXAS activity, serotonin release, and p^38 MAPK^ and ERK2 phosphorylation in collagen- and ADP-activated rat platelets without affecting blood coagulation. Furthermore, WIB-801CE manifested in vivo inhibitory effect on collagen plus epinephrine-induced pulmonary thromboembolism mice model. WIB-801CE inhibited in vitro NO production and fibrin clot retraction, but elevated free radical scavenging activity without affecting cytotoxicity against human platelets.

**Conclusion:**

WIB-801CE inhibited collagen- and ADP-induced platelet activation and its associated thrombus formation ex vivo and in vivo. These were resulted from down-regulation of TXA_2_ production and its related AA release and TXAS activity, and p^38^ ^MAPK^ and ERK2 activation. These results suggest that WIB-801CE has therapeutic potential to treat platelet activation-mediated thrombotic diseases in vivo.

## Background

**Fig. 1:**
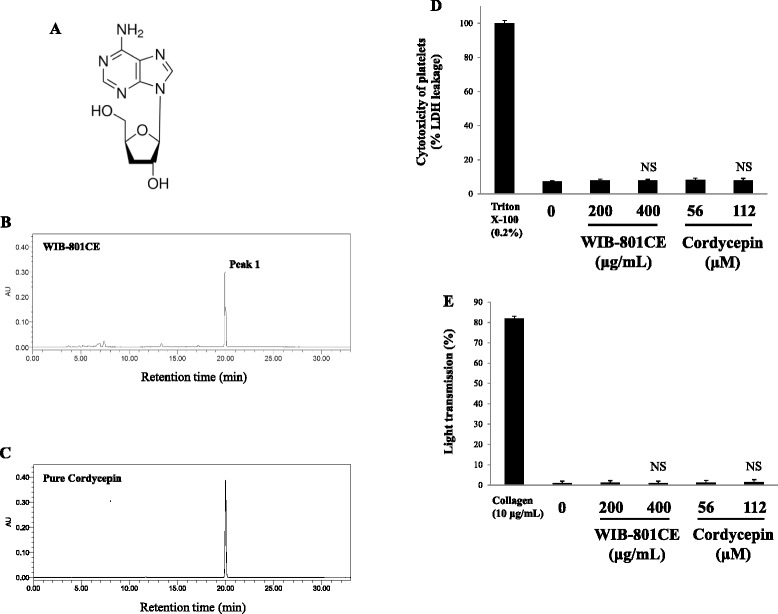
Composition of cordycepin in WIB-801CE and effects of cordycepin and WIB-801CE on cytotoxicity and platelet activation. **a** Chemical structure of cordycepin (3'-deoxyadenosine). **b** The chromatogram of WIB-801CE. **c** The chromatogram of pure cordycepin. **d** In vitro effects of WIB-801CE and cordycepin on cytotoxicity. **e** In vitro effects of WIB-801CE and cordycepin on platelet aggregation without agonists. Measurements of cordycepin, cytotoxicity, and platelet aggregation were carried out as described in “Methods” section. As a positive control to LDH cytotoxicity and platelet activation, 0.2% triton X-100 and collagen (10 μg/mL) were used, respectively. The data are expressed as the mean ± standard deviation (*n* = 4). NS, not significant versus without WIB-801CE and cordycepin, each control

A species of the fungal genus *Cordyceps* is known to prescribe for inflammatory and cancer disease [1, 2]. It is reported that cordycepin (3'-deoxyadenosine, Fig. 1a), a major component of *Cordyceps militaris*, has in vitro antithrombotic effects by attenuating [Ca^2+^]_i_ level and thromboxane A_2_ (TXA_2_) production in collagen-induced human platelet aggregation [3]. However, there is no evidence or report concerning ex vivo and in vivo inhibitory effect of cordycepin or cordycepin-enriched substance on platelet activation.

In this study, to resolve this doubtful point, we prepared cordycepin-enriched WIB-801CE (*C*ompound from 20*08 First* Project of *B*ioteam, *W*han*i*n Pharm. Co., Ltd., Suwon, Korea), an ethanol extract from *Cordyceps militaris*-hypha. Next, to observe whether WIB-801CE has endogenous inhibitory effects on platelet activation associated with thrombus formation, we orally administered WIB-801CE to rat, and subsequently investigated the effects on major molecules associated with Ca^2+^ increase [4–7], arachidonic acid (AA) release [4, 6, 8–10], TXA_2_ production [4, 5, 8, 11–13] and serotonin release [13–16].

## Methods

### Materials

WIB-801CE was provided from Whanin Pharmaceutical Corporation (Suwon, Korea). Collagen, adenosine diphosphate (ADP) and thrombin were obtained from Chrono-Log Corporation (Havertown, PA, USA). Serotonin enzyme-linked immunosorbent assay (ELISA) kit was purchased from Labor Diagnostika Nord GmbH & Corporation (Nordhorn, Germany). Pure cordycepin, aspirin, protease inhibitor cocktail, 2,2-diphenyl-1-picrylhydrazyl (DPPH), ascorbic acid (AC), and other reagents were obtained from Sigma Chemical Corporation (St. Louis, MO, USA). Thromboxane B_2_ (TXB_2_) enzyme immunoassay (EIA) kit, cyclooxygenase-1 (COX-1) fluorescent activity assay kit, lactate dehydrogenase (LDH) cytotoxicity assay kit, ozagrel and prostaglandin H_2_ (PGH_2_) for TXA_2_ synthase (TXAS) assay were purchased from Cayman Chemical (Ann Arbor, MI, USA). Arachidonic acid (AA) release ELISA kit was purchased from Cusabio Biotech Corporation (Wuhan, Hubei, China). Anti-phosphor-cytosolic phospholipase A_2_ (cPLA_2_) (Ser^505^), anti-phosphor-phospholipase C_β3_ (PLC_β3_) (Ser^537^), anti-phosphor-phospholipase C_β3_ (PLC_β3_) (Ser^1105^), anti-phosphor-phospholipase C_γ2_ (PLC_γ2_) (Tyr^1217^), anti-phosphor-p^38 MAPK^, anti-phosphor-extracellular signal–regulated kinase (ERK) (1/2), anti-p^38 MAPK^, anti-ERK (1/2) and anti-rabbit immunoglobulin G (IgG)-horseradish peroxidase conjugate (HRP), and lysis buffer were obtained from Cell Signaling (Beverly, MA, USA). Polyvinylidene difluoride (PVDF) membrane was from General Electric Healthcare (Piseataway, NJ, USA). Enhanced chemiluminescence solution (ECL) was from General Electric Healthcare (Chalfont St. Giles, Buckinghamshire, UK). Prothrombin time (PT) assay reagent and activated partial thromboplastin time (APTT) assay reagent were obtained from Fisher Diagnostics (Middletown, VA, USA).

### Preparation of WIB-801CE

*Cordyceps militaris* was cultivated, and culture-solution of *Cordyceps militaris*-hypha was concentrated up to 50° Brix with a rotary vacuum evaporator (Eyela N3000, Rikakikai Co., Ltd., Tokyo, Japan) at 60 °C. The Brix was measured with refractometer (Atago Co., Ltd., Tokyo, Japan). The concentrate was extracted by extraction-shaker (Cosmos 660, Kyungseo Co., Ltd., Seoul, Korea) for 4 h at 40 °C one time with distilled water/95% ethanol (1:3.5, v/v), which was filtered one time using a filter paper (Advantec No.2). The filtrate was completely concentrated at 60 °C by an evaporator (Eyela N3000, Rikakikai Co., Ltd., Tokyo, Japan) under reduced pressure, and was lyophilized and stored at -20 °C until used. This was named as cordycepin-enriched WIB-801CE.

### Analysis of cordycepin in WIB-801CE with HPLC

WIB-801CE was dissolved with 75% methanol, then analyzed by high performance liquid chromatography (HPLC). An Alliance 2695 liquid chromatography system (Waters Co., Milford, MA, USA), equipped with vacuum degasser, quaternary gradient pump, autosampler and photodiode array detector, was connected to Empower software. A hydrosphere C_18_ column (250 mm × 4.6 mm id, 5 μm, YMC Co., Ltd., Kyoto, Japan) was used at a column temperature of 30 °C. The applied-mobile phase gradient program was 0.01 M KH_2_PO_4_/methanol (95:5, v/v) at 0 min and held for 5 min; 0.01 M KH_2_PO_4_/methanol v/v) at 20 min and held for 6 min; 0.01 M KH_2_PO_4_/methanol (95:5, v/v) at 27 min and held 6 min for chromatographic balance. In this step, 99.8% of methanol was used. The flow rate was at 1.0 mL/min and sample injection volume was 10 μL. The ultra violet detection was operated at 254 nm.

### Animals and administration

We investigated the ex vivo and in vivo effects of WIB-801CE using rats (Sprague-Dawley, male, 200 g) and Institute of Cancer Research (ICR) mice (male, 18 g, Daehan Biolink Co., Ltd., Chungbook, Korea). Rats for ex vivo experiment and mice for in vivo observation were divided into as follows, respectively: WIB-801CE-nontreated group (control), WIB-801CE-treated group, aspirin-treated group as positive control of in vivo, and warfarin-treated group as positive control of ex vivo.

Animals were acclimatized for a week at a temperature of 24 ± 1 °C and humidity of 55 ± 5%. Before oral administration of substances, all animals were fasted for 12 h, then were fed with standard pellets diet (Purina Inc., Korea) had free access to water. WIB-801CE [200, 400 mg/kg-body weight (BW)] and warfarin (1 mg/kg-BW) for ex vivo experiment were orally administered to the rats one per day for seven days, and WIB-801CE (200, 400 mg/kg-BW) and aspirin (100 mg/kg-BW) for in vivo observation were orally administered to the mice once a day for five days. 200 mg/kg-BW of WIB-801CE is corresponded to the minimum dose that inhibits rat platelet aggregation (data not shown). WIB-801CE, warfarin, and aspirin were dissolved with distilled water. The experiments were proved by the Ethics Committee for Animal Experiments of Whanin Pharmaceutical Corporation (Suwon, Korea/15-NE-016 for rats, 15-NE-008 for mice).

### Preparation of rat platelet-rich plasma and platelet-poor plasma for ex vivo assay

After the final respective administration, all rats were fasted for 24 h, then after 2 h of WIB-801CE- and warfarin-administration were anesthetized with 20% urethane before sacrifice according to the method of Zhang et al. [17]. The blood was collected from the abdominal aorta. The blood was anti-coagulated with acid-citrate-dextrose solution (0.8% citric acid, 2.2% sodium citrate, 2.45% glucose), and was centrifuged at 250 × g for 10 min in order to obtain platelet-rich plasma (PRP). In order to remove residual red blood cells and white cells, the PRP was again centrifuged at 125 × g for 10 min. Platelet-poor plasma (PPP) was prepared by centrifuging the part of PRP at 1,300 × g for 10 min.

PRP was used to investigate ex vivo platelet aggregation, TXA_2_ production, serotonin release, COX-1 and TXAS activities, AA release and protein phosphorylation. PPP was used to investigate ex vivo PT and APTT. The number of platelets in PRP was adjusted with PPP to a final concentration of 5 × 10^8^/mL. All of the above procedures were carried out at 25 °C to avoid platelet aggregation from any effect of low temperature.

### Preparation of human PRP and washed platelets for in vitro assay

To investigate in vitro effects of WIB-801CE and cordycepin on fibrin clot retraction, we used human PRP and washed platelets. PRP from normal healthy human volunteers with informed consent was obtained from the Korean Red Cross Blood Center (KRBC, Changwon, Korea), and its experimental use was approved by the KRBC (Safety Supervisor Team-621-2015.02.26) and the Korea National Institute for Bioethics Policy Public Institutional Review Board (Seoul, Korea/PIRB12-072-01) with informed consent. PRP anticoagulated with acid-citrate-dextrose solution (0.8% citric acid, 2.2% sodium citrate, 2.45% glucose) was centrifuged for 10 min at 125 × g to remove a little red blood cells and white cells, which was used to investigate the effect of WIB-801CE and cordycepin on thrombin-induced fibrin clot retraction. The number of platelets in PRP was adjusted with PPP to a final concentration of 5 × 10^8^/mL.

To observe in vitro effects of WIB-801CE and cordycepin on cytotoxicity and resting platelet aggregation, we prepared human washed platelets. The PRP was centrifuged for 10 min at 1,300 × g to obtain platelet pellets. The platelets were washed twice with washing buffer (138 mM NaCl, 2.7 mM KCl, 12 mM NaHCO_3_, 0.36 mM NaH_2_PO_4_, 5.5 mM glucose, and 1 mM Na_2_EDTA, pH 6.5). The washed platelets were then resuspended in suspension buffer (138 mM NaCl, 2.7 mM KCl, 12 mM NaHCO_3_, 0.36 mM NaH_2_PO_4_, 0.49 mM MgCl_2_, 5.5 mM glucose, 0.25% gelatin, pH 6.9) to a final concentration of 5 × 10^8^/mL. All of the above procedures were carried out at 25 °C to avoid platelet aggregation from any effect of low temperature. The Korea National Institute for Bioethics Policy Public Institutional Review Board (Seoul, Korea/PIRB12-072-01) approved these experiments.

### In vitro cytotoxicity assay

Platelet cytotoxicity was determined by leakage of LDH from cytosol. Human washed platelets (10^8^/mL) were incubated for 5 min at 37 °C in the presence of WIB-801CE or cordycepin, then centrifuged with 12,000 × g at room temperature for 2 min. The supernatant was measured with a synergy HT multi-model microplate reader (BioTek Instruments, Winooski, VT, USA) using LDH assay kit. LDH leakage was expressed as percentage of total LDH activity in platelets completely lysed by 0.2% triton X-100.

### Measurement of ex vivo rat platelet aggregation, and in vitro human resting platelet aggregation

To evaluate antiplatelet effect of WIB-801CE under condition that generates maximally platelet aggregation, we used high dose of collagen and ADP as agonists. The concentration of collagen-induced maximal rat (Sprague-Dawley, male) platelet aggregation was 10 μg/mL [18], and 5 μM of ADP was used to aggregate rat platelets [19]. Accordingly, we used 10 μg/mL of collagen, and 5 μM of ADP to cause ex vivo rat platelet aggregation.

To measure ex vivo rat platelet aggregation, PRP (10^8^ platelets/mL) were preincubated with or without WIB-801CE for 3 min at 37 °C, then stimulated for 5 min by collagen (10 μg/mL) and ADP (5 μM) using an aggregometer (Chrono-Log Corporation, Havertown, PA, USA) at a constant stirring speed of 1,000 rpm.

To observe in vitro effects of WIB-801CE and cordycepin on resting human platelet aggregation, the aggregation of human washed platelets (10^8^/mL) was performed as described above in the presence of WIB-801CE or cordycepin without agonists. But, collagen (10 μg/mL) was used as positive control. Each aggregation rate was determined as an increase in light transmission. The PPP for PRP aggregation, and platelet suspension buffer (pH 6.9) for washed platelet aggregation were used as the reference (transmission 0) to regulate the base line of aggregometer. WIB-801CE and cordycepin were dissolved in distilled water.

### Ex vivo measurement of TXB_2_

To investigate the effect on TXA_2_ production, the aggregation was terminated by adding ice-cold 5 mM EDTA and 0.2 mM indomethacin to inhibit subsequent conversion of AA to TXA_2_. The amounts of TXB_2_, a stable metabolite of TXA_2_, were determined using a TXB_2_ EIA kit according to the procedure described by the manufacturer.

### Ex vivo Western blot for analysis of protein phosphorylation

Collagen- and ADP-activated rat PRP was centrifuged for 10 min at 1,300 × g under 4 °C to remove PPP and get platelet pellets. The platelets were suspended twice with suspension buffer (138 mM NaCl, 2.7 mM KCl, 12 mM NaHCO_3_, 0.36 mM NaH_2_PO_4_, 0.49 mM MgCl_2_, 5.5 mM glucose, 0.25% gelatin, pH 7.4). The suspended platelets (250 μL) were lysed by adding an equal volume (250 μL) of lysis buffer [20 mM Tris-HCl, 150 mM NaCl, 1 mM Na_2_EDTA, 1 mM EGTA, 1% triton X-100, 2.5 mM sodium pyrophosphate, 1 mM β-glycerophosphate (serine/threonine phosphatase inhibitor), 1 mM Na_3_VO_4_ (ATPase, alkaline and acid phosphatase, and protein phosphotyrosine phosphatase inhibitor), 1 μg/mL leupeptin (serine and cysteine protease inhibitor), and 1 mM phenylmethanesulfonyl fluoride (serine protease and acetylcholinesterase inhibitor), pH 7.5].

Platelet lysates were suspended in their equal volume of sodium dodecyl sulfate-polyacrylamide gel electrophoresis (SDS-PAGE) buffer (62.5 mM Tris-HCl, 10% glycerol, 1% SDS, 1% β-mercaptoethanol, 0.01% bromphenol blue, pH 6.8), then were boiled to completely denature the proteins for 5 min. Aliquots containing 15 μg of protein from each sample tube were subjected to SDS-PAGE (8%, 1.5 mm gel) according to the method of Laemmli [20].

Proteins in the gel were transferred to PVDF membrane in the presence of transfer buffer (25 mM Tris-HCl, 192 mM glycine, 20% methanol, pH 8.3). PVDF membrane was washed one time for 5 min with Tris-buffered saline with tween 20 (25 mM Tris-HCl, 140 mM NaCl, 2.7 mM KCl, 0.1% tween 20, pH 7.4), then was blocked with blocking buffer (25 mM Tris-HCl, 140 mM NaCl, 2.7 mM KCl, 0.1% tween 20, 5% skim milk, pH 7.4) for 1 h at room temperature, and subsequently was washed three times for 5 min.

The protein phosphorylation was observed using Western blotting. The dilutions for 1st antibody (anti-phosphor-cPLA_2_, anti-phosphor-PLC_β3_, anti-phosphor-PLC_γ2_, anti-phosphor-p^38 MAPK^, anti-phosphor-ERK, anti-p^38 MAPK^, anti-ERK) and 2nd antibody (anti-rabbit IgG-HRP) were 1:1,000 and 1:10,000, respectively. The membranes were visualized using ECL. Blots were analyzed using the Quantity One, Ver. 4.5 (BioRad, Hercules, CA, USA).

### Ex vivo determination of AA release

To investigate the effect on AA release, the aggregation was terminated adding ice-cold 5 mM EDTA and 0.2 mM indomethacin to inhibit subsequent conversion of AA to TXA_2_, and centrifuged with 200 × g at 4 °C for 10 min. The supernatants were used for the assay of AA release. AA release was measured with a Synergy HT multi-model microplate reader (BioTek Instruments, Winooski, VT, USA) using AA release ELISA kit.

### Preparation of platelet lysates

We prepared platelet lysates to determine ex vivo COX-1 and TXAS activities. Collagen- and ADP-activated rat PRP was centrifuged for 10 min at 1,300 × g to remove PPP and get platelet pellets. The platelets were then suspended twice with suspension buffer (138 mM NaCl, 2.7 mM KCl, 12 mM NaHCO_3_, 0.36 mM NaH_2_PO_4_, 0.49 mM MgCl_2_, 5.5 mM glucose, 0.25% gelatin, pH 7.4). The suspended platelets in the presence of 1% protease inhibitor cocktail were sonicated ten times in sensitivity 100% for 20 s at 4 °C with a sonicator (HD 2070, Bandelin Electronic, Bandelin, Germany) to obtain platelet lysates. Next, the platelet lysates were centrifuged at 12,000 × g for 15 min at 4 °C to remove cell debris. The supernatant was used to measure COX-1 and TXAS activity.

### Ex vivo measurement of COX-1 activity

Platelet lysates containing 10 μg of protein were used. COX-1 activity was measured with a Synergy HT multi-model microplate reader (BioTek Instruments, Winooski, VT, USA) using COX-1 fluorescent activity assay kit according to the procedure described by manufacturer.

### Ex vivo measurement of TXAS activity

Platelet lysates containing 20 μg of protein were used. The reaction for assay of TXAS activity was initiated by the addition of TXAS substrate PGH_2_ and allowed to proceed for 1 min at 37 °C. The reaction was terminated by the addition of 1 M citric acid, then was neutralized with 1 N NaOH. The concentration of TXA_2_ was determined as TXB_2_, a stable metabolite of TXA_2_, which was measured with a Synergy HT multi-model microplate reader (BioTek Instruments, Winooski, VT, USA) using TXB_2_ EIA kit.

### Ex vivo determination of serotonin release

To investigate the effect on serotonin release, the aggregation was centrifuged at 4 °C for 10 min at 200 × g. The supernatants were used for the assay of serotonin release. Serotonin release was measured with a Synergy HT multi-model microplate reader (BioTek Instruments, Winooski, VT, USA) using serotonin ELISA kit.

### Ex vivo measurement of PT and APTT

To investigate whether WIB-801CE shows anticoagulant characteristics, if any, has bleeding risk as side effect of anticoagulant [21], we measured PT and APTT, markers of blood coagulation. The PPP (100 μL) was preincubated in a two-channel coagulator (Behnk Elektronik GmbH & Co., KG, Norderstedt, Germany) cup (catalog number 95-662, BioMérieux, Marcyl’Etoile, France) with gentle stirring for 1 min at 37 °C. PT was determined as the time interval between the addition of PT reagent (100 μL) to the PPP and the formation of a fibrin clot. After preincubation of PPP for APTT measurement, 100 μL of APTT reagent was added to the PPP (100 μL) and incubated for 3 min at 37 °C. Following incubation, 100 μL of 25 mM CaCl_2_ was immediately added to the PPP containing APTT reagent. APTT was determined as the time required to form a fibrin clot.

### In vivo tail bleeding time assay

We investigated whether WIB-801CE has bleeding risk, the side effect of antiplatelet substance [21]. WIB-801CE (200, 400 mg/kg-BW) and aspirin (100 mg/kg-BW), a positive control, were orally administered to mice once a day for five days. In this study, we used mice for measuring tail bleeding time according to the method of Kim and Lee [22]. After 5 min of the respective final administration, mice were anesthetized with zoletil (40 mg/kg, i.p.). The distal 0.5 cm segment of the tail was transected with operating knife, and immediately immersed in a tube containing 37 °C of saline. Tail bleeding time was determined as the time required to cause blood coagulation.

### In vivo evaluation of anti-acute pulmonary thromboembolism

To confirm the endogenous antithrombotic effect, we used a mice model to generate acute pulmonary thromboembolism [23]. Mice were orally administered with WIB-801CE (200, 400 mg/kg-BW), and aspirin (100 mg/kg-BW). After 1 h of respective administration, the mixture (100 μL) of collagen (300 μg/kg-BW) plus epinephrine (30 μg/kg-BW) were injected *via* tail vein, and the rate of protection and mortality was observed for 15 min, which were calculated as follow: 1) Protection rate (%) = [(Number of tested mice – Number of dead mice)/Number of tested mice] × 100. 2) Mortality rate (%) = [Number of dead mice/Number of tested mice] × 100. These experiments were proved by the Ethics Committee for animal experiments of Whanin Pharmaceutical Corporation (Suwon, Korea/15-NE-009).

### In vitro assay of platelet-mediated fibrin clot retraction

We investigated whether WIB-801CE or cordycepin inhibits fibrin clot retraction, an index of thrombi formation [24]. Human PRP 250 μL (10^8^ platelets/mL) were transferred into polyethylene tube to avoid clot adherence, then were preincubated with or without WIB-801CE or cordycepin for 10 min at 37 °C, and subsequently stimulated with thrombin (0.5 U/mL) for 60 min at 37 °C. Pictures of fibrin clot were taken at 0 and 60 min using a digital camera, and its quantification was carried out by measurement of clot area using the NIH Image J Software (V1.46, National Institutes of Health, USA). Percentage of clot retraction was calculated as follows: Retraction (%) by thrombin = [1 - (final clot area/initial clot area)] × 100.

### In vitro nitric oxide assay

To observe antiinflammatory effect of WIB-801CE, we used mouse leukemic macrophage RAW264.7 cells. RAW264.7 cells were obtained from the American Type Culture Collection (ATCC, Manassas, VA, USA), and were maintained at 37 °C in 5% CO_2_ and 95% air in Dulbecco’s Modified Eagle’s Medium (GE Healthcare, Marlborough, USA) containing 10% fetal bovine serum, and 1% penicillin-streptomycin solution. RAW264.7 cells (5 × 10^4^ cells) were preincubated for 30 min with or without WIB-801CE, or inducible nitric oxide synthase (iNOS) inhibitor amino guanidine (AG), and stimulated for 24 h by lipopolysaccharide (10 ng/mL). The supernatant was used for NO assay using Griess reagent. Equal volume of culture supernatant (80 μL) and Griess reagent (80 μL) were mixed. The absorbance of the mixture was measured at 540 nm using spectrophotometer (Spectramax 190, Molecular devices, LLC., Sunnyvale, CA, USA). Nitrite was used as standard of NO.

### Ex vivo nitric oxide assay

To investigate the NO production, we used collagen- and ADP-stimulated PRP obtained from rats administered with the WIB-801CE (200, 400 mg/kg-BW). The PRP was centrifuged at 4 °C for 10 min at 10,000 × g to get plasma. The plasma was incubated 1 h with 400 μL methanol:diethylether (3:1 mixture v/v), and subsequently plasma proteins were precipitated by centrifuging at 4 °C for 10 min at 10,000 × g. The supernatant was used for NO assay using Griess reagent. Equal volume of supernatant (80 μL) and Griess reagent (80 μL) were mixed. After 30 min, absorbance of the mixture was measured at 540 nm using a Synergy HT multi-model microplate reader (BioTek Instruments, Winooski, VT, USA). Nitrite was used as standard of NO.

### In vitro determination of antioxidant activity

To obtain antioxidant effect of WIB-801CE, we measured scavenging activity of free radical in DPPH according to the method [25, 26]. DPPH was dissolved in 99% ethanol to make 200 μM of solution. WIB-801CE and antioxidant AC were dissolved in distilled water. Equal volume of test substances and DPPH were mixed at room temperature. After 30 min, the reduction in DPPH absorbance at 517 nm was measured using spectrophotometer (Optizen 2120UV, Mecasys, Korea). The scavenging activity of DPPH radicals by substances was determined using the following equation [26]: Scavenging activity (%) = [1 – (A_sample_/A_DPPH_)] × 100. The absorbance at 517 nm by 99% ethanol, DPPH vehicle, and distilled water, vehicles of WIB-801CE and AC was 0.001 and 0.000.

### Ex vivo measurement of cordycepin effect on rat platelet aggregation

This experiment was performed to investigate the effect of cordycepin on ex vivo platelet aggregation. When cordycepin (15 mg/kg per day) was administered orally to the mice for 14 days, antitumor activity was known to observe [27]. Therefore, we selected 5 and 10 mg/kg-BW per day of cordycepin in this experiment as moderate doses for administration. These doses are corresponded to about 36 and 72% of cordycepin in WIB-801CE (200 mg/kg-BW) that inhibited ex vivo rat platelet aggregation. Rats (Sprague-Dawley, male, 200 g) were acclimatized for a week at a temperature of 24 ± 1 °C and humidity of 55 ± 5%. Before oral administration of cordycepin, rats were fasted for 12 h, then were fed with standard pellets diet (Purina Inc., Korea) had free access to water. Cordycepin (5 and 10 mg/kg-BW) was orally administered to the rats one per day for seven days. Cordycepin were dissolved with distilled water. The experiments were proved by the Ethics Committee for Animal Experiments of Whanin Pharmaceutical Corporation (Suwon, Korea/15-NE-016 for rats). After the final respective administration, all rats were fasted for 24 h, then were anesthetized with 20% urethane before sacrifice. PRP preparation, platelet aggregation, measurement were performed as described before.

### Protein assay

To determine COX-1, TXAS activity, and protein phosphorylation, protein concentration was measured using bicinchoninic acid assay kit (Pierce Biotechnology, USA).

### Statistical analyses

The experimental results are indicated as the mean ± standard deviation accompanied by the number of observations. Data were determined by analysis of variance (ANOVA). If this analysis showed significant differences among the group means, then each group was compared by the Newman-Keuls method. Statistical analysis was carried out according to the SPSS 21.0.0.0 (SPSS, Chicago, IL, USA). *p* < 0.05 was considered to be statistically significant.

## Results

### Composition of cordycepin in WIB-801CE

Because it is known that *Cordyceps militaris*, a source of WIB-801CE, has cordycepin (Fig. 1a) [28], we analyzed cordycepin of WIB-801CE with HPLC. As shown in Fig. 1b, peak 1 from WIB-801CE was observed at 19.988 min of the retention time, which was almost in accord with the retention time (19.980 min) of pure cordycepin (Fig. 1c). This means that peak 1 is derived from cordycepin in WIB-801CE. The concentration of peak 1 in WIB-801CE corresponding to cordycepin was 69.30 ± 0.20 mg/g-WIB-801CE (about 6.93 ± 0.02%, Table 1). Whole fruiting body myelia of *Cordyceps militaris* is known to contain 0.16% of cordycepin, but whole fruiting body, stroma, and larva of *Cordyceps sinensis* do not contain cordycepin [29]. Therefore, the cordycepin content in WIB-801CE that we used in this study is very higher than those in whole fruiting body myelia of *Cordyceps militaris*, and in whole fruiting body, stroma, and larva of *Cordyceps sinensis*.

**Table 1:** Content of cordycepin in WIB-801CE

	Retention time (min)	Area (mAU × s)	Concentration of sample (μg/mL)	Cordycepin content (%)	Content (mg/g-WIB-801CE)
Pure cordycepin	19.980	2,949.35	100.30	-	-
WIB-801CE Peak 1	19.988 ± 0.011	2,278.34 ± 13.77	1,006 ± 0.32	6.93 ± 0.02	69.30 ± 0.20

The content of cordycepin in WIB-801CE was expressed using the following equation: Cordycepin content (%) = (area of peak 1/area of pure cordycepin) × (concentration of pure cordycepin/concentration of WIB-801CE) × [(100% - % of water content of cordycepin)/100%] × (% of purity of pure cordycepin/100%) × 100%. Water content of pure cordycepin was 8.18%. Purity of pure cordycepin was 98.0%. The data are given as the mean ± standard deviation (*n* = 3)

### In vitro effects of WIB-801CE and cordycepin on cytotoxicity against resting human platelets

Cytotoxicity of drugs or substances is evaluated by cytosolic LDH leakage, which is different from platelet aggregation or granule secretion by platelet agonists, but is released upon damage of cell membrane [30–32]. Therefore, we investigated the effect of WIB-801CE on LDH leakage to human platelets. When human washed platelets (10^8^/mL) were treated by membrane detergent triton X-100, LDH was potently released up to 268.6 ± 4.4 mU/10^8^ platelets, which is expressed as 100% (Fig. 1d). LDH leakage by WIB-801CE (200, 400 μg/mL) was not occurred, and its degree (8.6 ± 1.4%) by 400 μg/mL of WIB-801CE was not significantly different from that (7.3 ± 0.4%) by resting platelets (Fig. 1d). Because WIB-801CE contains 6.93 ± 0.02% of cordycepin (Table 1), to investigate whether cordycepin has cytotoxicity, we used concentrations (56, 112 μM) of cordycepin (MW 251.14) corresponding to cordycepin concentration (6.93 ± 0.02%) that contains in WIB-801CE (200, 400 μg/mL). As the results, cordycepin (56, 112 μM) did not affect the release of LDH as compared with that (7.3 ± 0.4%) by resting platelets (Fig. 1d). These mean that WIB-801CE and cordycepin do not affect the cytotoxicity to human resting platelets.

### In vitro effects of WIB-801CE and cordycepin on activation of resting human platelets

Platelet activation is an index of platelet shape change, platelet aggregation and granule secretion, and is the cause of cardiovascular and cerebrovascular disease, and atherosclerosis [33–35]. Accordingly, if WIB-801CE activates resting platelets, unstimulated platelets, a question to evaluate the antiplatelet effects of WIB-801CE might be raised. Therefore, the effect of WIB-801CE and cordycepin on platelet activation was determined by measuring platelet aggregation in resting human platelets. As the results, a positive control collagen (10 μg/mL) activated platelets by increasing platelet aggregation up to 83.3 ± 3.1% (Fig. 1e). However, WIB-801CE (200, 400 μg/mL) alone, and cordycepin (56, 112 μM) alone did not increased platelet aggregation (Fig. 1e), as compared with that (1.0 ± 1.0%) by resting platelets. It was evidenced that WIB-801CE and cordycepin alone do not affect the activation of resting human platelets.

### Ex vivo effects of WIB-801CE on platelet aggregation and TXA_2_ production

**Fig. 2:**
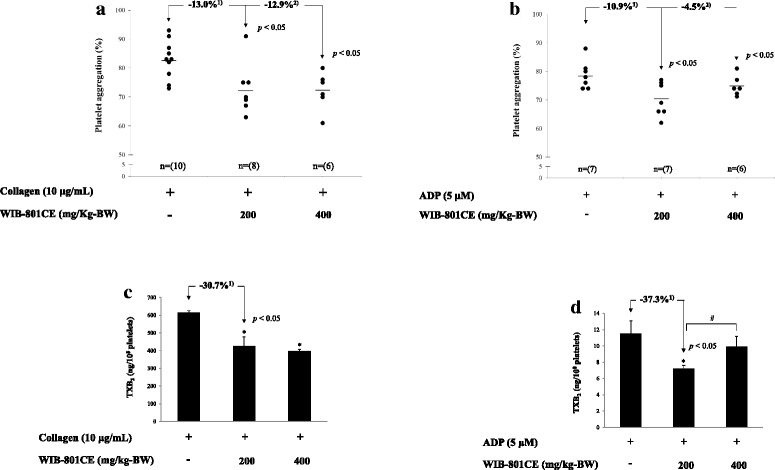
Ex vivo effects of WIB-801CE on platelet aggregation and TXB_2_ production. **a** Ex vivo effects of WIB-801CE on collagen-induced platelet aggregation. **b** Ex vivo effects of WIB-801CE on ADP-induced platelet aggregation. **c** Ex vivo effects of WIB-801CE on collagen-induced TXB_2_ production. **d** Ex vivo effects of WIB-801CE on ADP-induced TXB_2_ production. Measurements were carried out as described in “Methods” section. The data are expressed as the mean ± standard deviation (*n* = 6 to 10). ^*^ *p* < 0.05 versus the each agonist-stimulated platelets, ^#^ *p* < 0.05 versus the ADP-stimulated platelets in the presence of WIB-801CE 200 mg/kg-BW. ^1)^ Δ (%) = [(agonist + WIB-801CE 200 mg/kg-BW) – agonist]/agonist × 100, ^2)^ Δ (%) = [(agonist + WIB-801CE 400 mg/kg-BW) – agonist]/agonist × 100

It is known that the inhibition of collagen- and ADP-induced platelet aggregation is potential target to develop antithrombotic agent having antiplatelet characteristics [36, 37]. Therefore, we used on collagen and ADP as agonists. When PRP (10^8^/mL) from control was activated with collagen and ADP, the aggregation rate was increased up to 82.9 ± 6.6% by collagen (Fig. 2a) and 78.7 ± 4.9% by ADP (Fig. 2b). However, collagen- and ADP-induced rat platelet aggregation was significantly attenuated by WIB-801CE (200, 400 mg/kg-BW) (Fig. 2a and b). The inhibitory degrees by 200 mg/kg-BW were 13.0% to that by collagen (Fig. 2a) and 10.9% against that by ADP (Fig. 2b).

We investigated next whether WIB-801CE involves in inhibition of TXA_2_ production to attenuate collagen- and ADP-induced rat platelet aggregation. When PRP (10^8^/mL) from control was activated by collagen and ADP, the TXA_2_ (determined as TXB_2_, a stable metabolite of TXA_2_) was increased to 615.0 ± 11.5 ng/10^8^ platelets (Fig. 2c) by collagen and 11.5 ± 1.6 ng/10^8^ platelets by ADP (Fig. 2d). However, collagen- and ADP-produced TXA_2_ were inhibited by WIB-801CE (Fig. 2c and d). The inhibitory degrees by 200 mg/kg-BW were 30.7% against that by collagen (Fig. 2c) and 37.3% to that by ADP (Fig. 2d). WIB-801CE (400 mg/kg-BW) inhibited weakly ADP-induced platelet aggregation (Fig. 2b) and TXA_2_ production (Fig. 2d) as compared with those by WIB-801CE (200 mg/kg-BW).

### Ex vivo effects of WIB-801CE on phosphorylation of PLC_β3_, PLC_γ2_, cPLA_2_, release of AA, and activity of COX-1 and TXAS

**Fig. 3:**
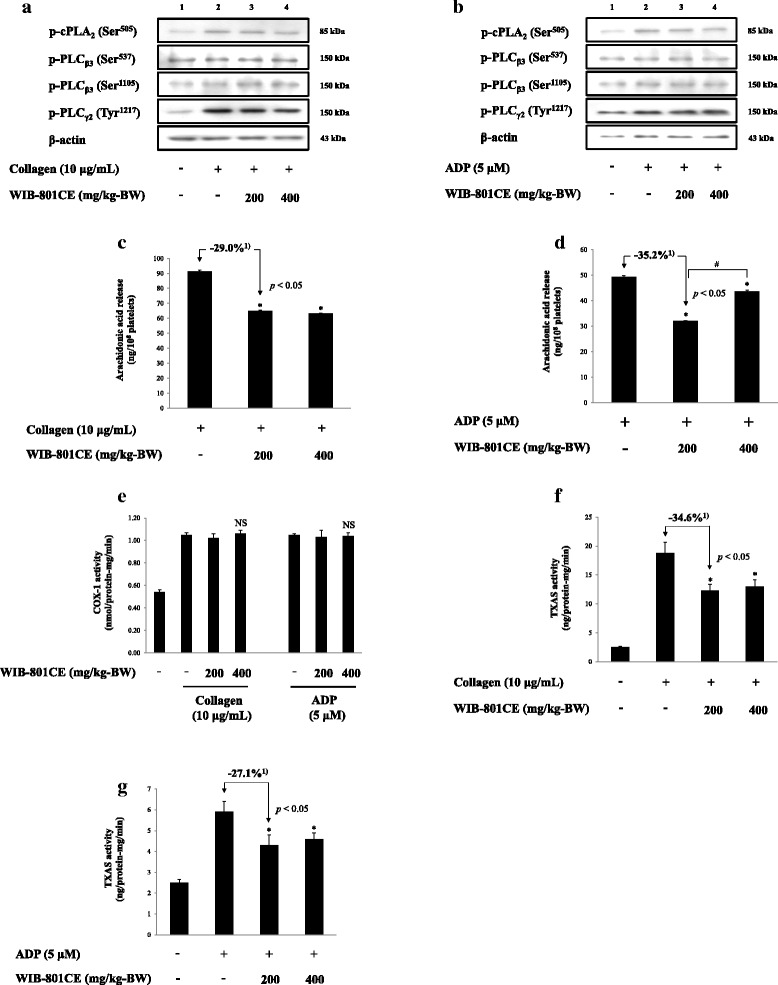
Ex vivo effects of WIB-801CE on phosphorylation of cPLA_2_, PLC_β3,_ PLC_γ2_, release of AA, and activity of COX-1 and TXAS. **a** Ex vivo effects of WIB-801CE on collagen-induced phosphorylation of cPLA_2_, PLC_β3_ and PLC_γ2_. Lane 1, unstimulated platelets; Lane 2, collagen (10 μg/mL); Lane 3, collagen (10 μg/mL) + WIB-801CE (200 mg/kg-BW); Lane 4, collagen (10 μg/mL) + WIB-801CE (400 mg/kg-BW). **b** Ex vivo effects of WIB-801CE on ADP-induced phosphorylation of cPLA_2_, PLC_β3_ and PLC_γ2_. Lane 1, unstimulated platelets; Lane 2, ADP (5 μM); Lane 3, ADP (5 μM) + WIB-801CE (200 mg/kg-BW); Lane 4, ADP (5 μM) + WIB-801CE (400 mg/kg-BW). **c** Ex vivo effects of WIB-801CE on collagen-induced arachidonic acid release. **d** Ex vivo effects of WIB-801CE on ADP-induced arachidonic acid release. **e** Ex vivo effects of WIB-801CE on COX-1 activity **f** Ex vivo effects of WIB-801CE on collagen-induced TXAS activity. **g** Ex vivo effects of WIB-801CE on ADP-induced TXAS activity. Measurements were carried out as described in “Methods” section. The data are expressed as the mean ± standard deviation (*n* = 4). ^*^ *p* < 0.05 versus each agonist-stimulated platelets. NS, not significant versus the each agonist-stimulated platelets, ^#^ *p* < 0.05 versus the ADP-stimulated platelets in the presence of WIB-801CE 200 mg/kg-BW. ^1)^ Δ (%) = [(agonist + WIB-801CE 200 mg/kg-BW) – agonist]/agonist × 100

The reduction of TXA_2_ by WIB-801CE is connected to either inhibition of AA release enzymes (PLC_β_, PLC_γ_ and cPLA_2_) or AA utilization enzymes (COX-1, TXAS). When PRP (10^8^/mL) from WIB-801CE (200, 400 mg/kg-BW) rats were activated by collagen and ADP, phosphorylation of PLC_β3_ (Ser^1105^), PLC _γ2_ (Tyr^1217^) and cPLA_2_ (Ser^505^) were increased (Fig. 3a and b, lane 2), but PLC_β3_ (Ser^537^) was not changed as compared with those (Fig. 3a and b, lane 1) by resting platelets. WIB-801CE did not inhibit these phosphorylation (Fig. 3a and b, lane 3 and 4). These results are indicated that WIB-801CE does not attenuate collagen- and ADP-induced the activities of cPLA_2_, PLC_β3_ and PLC_γ2_, and the direct AA release by cPLA_2_, but may involve in inhibition of the indirect AA release by PLC_β3_/diacylglycerol (DG)- and monoacylglycerol-lipase pathway [4, 5, 11, 12].

With this possibility, we investigated whether WIB-801CE affects AA release. Collagen and ADP released AA up to 91.5 ± 0.7 ng/10^8^ platelets (Fig. 3c) and 49.4 ± 0.4 ng/10^8^ platelets (Fig. 3d) in control, respectively. However, when PRP (10^8^/mL) from WIB-801CE (200, 400 mg/kg-BW) rats were activated by collagen and ADP, the levels of AA release were significantly decreased as compared with those by control (Fig. 3c and d). The inhibitory degrees by 200 mg/kg-BW were 29.0% (Fig. 3c) against that by collagen and 35.2% (Fig. 3d) against that by ADP.

WIB-801CE did not completely inhibit AA release (Fig. 3c and d), which means that collagen- and ADP-released AA may be subsequently metabolized to TXA_2_ *via* COX-1/TXAS pathway. Therefore, we investigated whether WIB-801CE inhibits ex vivo activity of COX-1 or TXAS. Collagen- and ADP-induced COX-1 activities as compared with those by resting platelets, but these were not attenuated by WIB-801CE (200, 400 mg/kg-BW) (Fig. 3e). Collagen and ADP induced TXAS activities as compared with those by resting platelets (Fig. 3f and g). However, collagen-induced TXAS activity (18.8 ± 1.9 ng/protein-mg/min) was reduced to 34.6% (12.3 ± 1.1 ng/protein-mg/min) by WIB-801CE (200 mg/kg-BW) (Fig. 3f). ADP-induced TXAS activity (5.9 ± 0.5 ng/protein-mg/min) was also attenuated to 27.1% (4.3 ± 0.5 ng/protein-mg/min) by WIB-801CE (200 mg/kg-BW) (Fig. 3g). WIB-801CE (400 mg/kg-BW) inhibited weakly ADP-induced AA release (Fig. 3d) and TXAS activity (Fig. 3g) as compared with those by WIB-801CE (200 mg/kg-BW).

### Ex vivo effects of WIB-801CE on serotonin release

**Fig. 4:**
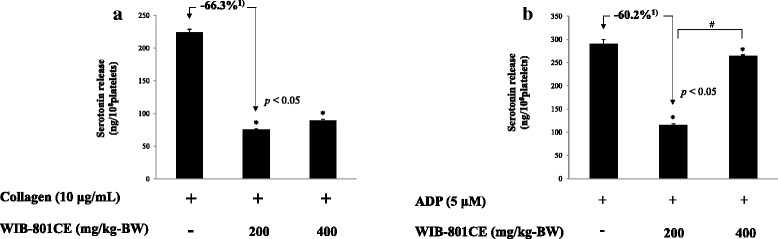
Ex vivo effects of WIB-801CE on serotonin release. **a** Ex vivo effects of WIB-801CE on collagen-induced serotonin release. **b** Ex vivo effects of WIB-801CE on ADP-induced serotonin release. Measurements were carried out as described in “Methods” section. The data are expressed as the mean ± standard deviation (*n* = 4). ^*^ *p* < 0.05 versus the each agonist-stimulated platelets, ^#^ *p* < 0.05 versus the ADP-stimulated platelets in the presence of WIB-801CE 200 mg/kg-BW. ^1)^ Δ (%) = [(agonist + WIB-801CE 200 mg/kg-BW) – agonist]/agonist × 100

Next, we investigated the effect of WIB-801CE on serotonin release as an index of granule secretion. Collagen and ADP elevated serotonin release up to 224.5 ± 4.3 ng/10^8^ platelets (Fig. 4a) and 290.1 ± 9.6 ng/10^8^ platelets (Fig. 4b), respectively. On the contrary, collagen- and ADP-released serotonin levels were reduced by WIB-801CE (200, 400 mg/kg-BW) (Fig. 4a and b). The inhibitory degrees by 200 mg/kg-BW were 66.3% to that by collagen (Fig. 4a) and 60.2% against that by ADP (Fig. 4b). WIB-801CE (400 mg/kg-BW) weakly inhibited ADP-induced serotonin release (Fig. 4b) as compared with that by WIB-801CE (200 mg/kg-BW).

### Ex vivo effects of WIB-801CE on p^38^^MAPK^- and ERK-phosphorylation

It is well reviewed that platelets contain MAPKs such as p^38^, ERK (1/2), and c-Jun N-terminal kinase [38]. These are activated by various agonists (i.e. collagen, ADP, thrombin, von Willebrand factor, fibrinogen) and subsequently stimulate various enzymes [myosin light chain kinase (MLCK), DG-lipase, cPLA_2_, COX-1] associated with platelet activation [9, 10, 38–46]. Of MAPKs, p^38^ ^MAPK^ and ERK2 stimulate MLCK [38–41] to release serotonin, DG-lipase [45] and cPLA_2_ [9, 10, 38, 42, 43] to produce AA, TXA_2_ precursor. In this study, to observe the relationship with the results that WIB-801CE inhibited collagen- and ADP-induced TXA_2_ production (Fig. 2c and d), AA release (Fig. 3c and d) and serotonin release (Fig. 4a and b), we investigated whether WIB-801CE inhibits the phosphorylation of p^38^ ^MAPK^ and ERK.

**Fig. 5:**
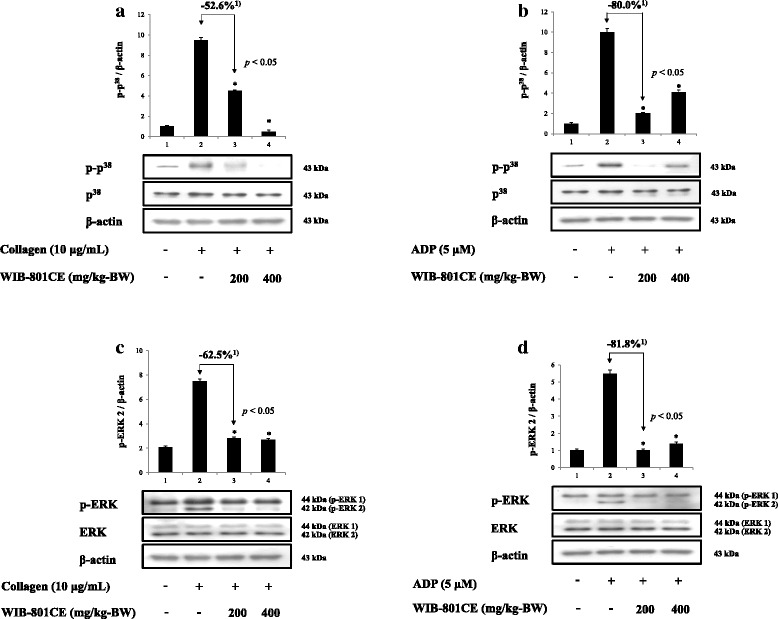
Ex vivo effects of WIB-801CE on phosphorylation of p^38 MAPK^ and ERK2. **a** Ex vivo effects of WIB-801CE on collagen-induced phosphorylation of p^38^ ^MAPK^. Lane 1, unstimulated platelets; Lane 2, collagen (10 μg/mL); Lane 3, collagen (10 μg/mL) + WIB-801CE (200 mg/kg-BW); Lane 4, collagen (10 μg/mL) + WIB-801CE (400 mg/kg-BW). **b** Ex vivo effects of WIB-801CE on ADP-induced phosphorylation of p^38 MAPK^. Lane 1, unstimulated platelets; Lane 2, ADP (5 μM); Lane 3, ADP (5 μM) + WIB-801CE (200 mg/kg-BW); Lane 4, ADP (5 μM) + WIB-801CE (400 mg/kg-BW). **c** Ex vivo effects of WIB-801CE on collagen-induced phosphorylation of ERK2. Lane 1, unstimulated platelets; Lane 2, collagen (10 μg/mL); Lane 3, collagen (10 μg/mL) + WIB-801CE (200 mg/kg-BW); Lane 4, collagen (10 μg/mL) + WIB-801CE (400 mg/kg-BW). **d** Ex vivo effects of WIB-801CE on ADP-induced phosphorylation of ERK2. Lane 1, unstimulated platelets; Lane 2, ADP (5 μM); Lane 3, ADP (5 μM) + WIB-801CE (200 mg/kg-BW); Lane 4, ADP (5 μM) + WIB-801CE (400 mg/kg-BW). Western blotting was performed as described in “Methods” section. The data are expressed as the mean ± standard deviation (*n* = 4). *p < 0.05 versus the each agonist-stimulated platelets. ^1)^ Δ (%) = [(agonist + WIB-801CE 200 mg/kg-BW) – agonist]/agonist × 100

Collagen and ADP increased potently p^38^ ^MAPK^ phosphorylation (Fig. 5a and b, lane 2) as compared with those by resting platelets (Fig. 5a and b, line 1), respectively. However, these were diminished by WIB-801CE (200, 400 mg/kg-BW) (Fig. 5a and b, lane 3, 4). WIB-801CE (200 mg/kg-BW) inhibited p^38^ ^MAPK^ phosphorylation up to 52.6% against that by collagen (Fig. 5a, lane 3) and 80.0% against that by ADP (Fig. 5b, lane 3).

Collagen and ADP elevated potently ERK2 (42 kDa) phosphorylation (Fig. 5c and d, lane 2) as compared with those by resting platelets (Fig. 5c and d, line 1), respectively. However, these were also vanished by WIB-801CE (200, 400 mg/kg-BW) (Fig. 5c and d, lane 3, 4). WIB-801CE (200 mg/kg-BW) inhibited ERK2 (42 kDa) phosphorylation up to 62.5% against that by collagen (Fig. 5c, lane 3) and 81.8% against that by ADP (Fig. 5d, lane 3). WIB-801CE (400 mg/kg-BW) inhibited weakly phosphorylation of both p^38 MAPK^ and ERK2 (Fig. 5b and d, lane 4) as compared with those (Fig. 5b and d, lane 3) by WIB-801CE (200 mg/kg-BW) in ADP-activated platelets.

### Ex vivo and in vivo effects of WIB-801CE on blood coagulation and tail bleeding time

Bleeding is connected to the attenuation of platelet aggregation and blood coagulation, and the inhibition of thrombosis [8, 21, 47, 48]. Accordingly, we investigated the effects of WIB-801CE on ex vivo blood coagulation time (PT, APTT) and in vivo tail bleeding time as indexes of bleeding. As shown in Table 2, both ex vivo PT and APTT were not significantly prolonged by PPP from WIB-801CE rats as compared with those (PT, 13.2 ± 2.3 s; APTT, 26.2 ± 4.1 s) by normal. Warfarin (1 mg/kg-BW) infinitely prolonged PT and APTT (Table 2).

**Table 2:** Ex vivo effects of WIB-801CE-administration on blood coagulation

Animal group	Dose (mg/kg-BW)	PT (s)	APTT (s)	N
Normal	N.D.	13.2 ± 2.3	26.2 ± 4.1	8
WIB-801CE	200	17.7 ± 4.9^NS^	26.3 ± 3.9^NS^	8
	400	16.8 ± 3.2^NS^	26.0 ± 3.9^NS^	8
Warfarin	1	∞	∞	6

The results were expressed as the mean ± standard deviation (*n* = 8 or 6). ∞, no coagulation; *NS* not significant versus normal; *N.D* normal diet, *N* number of tested rats; *BW* body weight, *PT* prothrombin time, *APTT* activated partial thromboplastin time *s*, second

With regard to the effects of WIB-801CE on tail bleeding time, WIB-801CE (200, 400 mg/kg-BW) significantly prolonged from 125.3 ± 17.0 s by control to 264.8 ± 79.0 s and 360.3 ± 83.8 s, respectively (Table 3). Aspirin (100 mg/kg-BW) also prolonged tail bleeding time to 1,800.0 ± 0.0 s (Table 3). Aspirin (100 mg/kg-BW) potently prolonged bleeding time to 1,336.5%, on the other hand, WIB-801CE (200, 400 mg/kg-BW) prolonged it up to 111.4% and 187.5% as compared with that (125.3 ± 17.0 s) by control, respectively (Table 3).

**Table 3:** In vivo effects of WIB-801CE on tail bleeding time

Animal group	Dose (mg/kg-BW)	Tail bleeding time (s)	Δ (%)	N
Control	Saline	125.3 ± 17.0	0	8
WIB-801CE	200	264.8 ± 79.0^*^	111.3	8
	400	360.3 ± 83.8^**^	187.5	8
Aspirin	100	1,800.0 ± 0.0^**^	1,336.5	5

The results were expressed as the mean ± standard deviation (*n* = 8 or 5). ^*^ *p* < 0.05 compared with control, ^**^ *p* < 0.001 compared with control. *N* number of tested mice, *BW* body weight, *s*, second Δ (%) = [(WIB-801CE or aspirin) control]/control × 100

### In vivo effects of WIB-801CE on acute pulmonary thromboembolism

Because antiplatelet drugs play an important role in protection of thrombus formation, we investigated whether WIB-801CE, inhibiting ex vivo platelet aggregation, has also a protective effect on endogenous thrombus formation. In this study, in vivo venous antithrombotic effect of WIB-801CE was estimated using collagen plus epinephrine-induced acute pulmonary thromboembolism mouse model [23, 49–51]. As shown in Table 4, when the mixture of collagen plus epinephrine was treated to mice, the protection rate was 4.2% against acute pulmonary thromboembolism, and the mortality rate was 95.8%. However, in WIB-801CE-treated mice, the protection degree from a pulmonary thromboembolism was increased to 25.0% by WIB-801CE (200 mg/kg-BW), and 35.0% by WIB-801CE (400 mg/kg-BW) in a dose dependent manner (Table 4). In aspirin (100 mg/kg-BW)-treated mice, the protection degree from a pulmonary thromboembolism was increased up to 35.0%, and the mortality was decreased to 65.0%, which were equal to those by WIB-801CE (400 mg/kg-BW)-treatment (Table 4). These mean that WIB-801CE is actually valid to protect venous thromboembolism like aspirin.

**Table 4:** In vivo effects of WIB-801CE on acute pulmonary thromboembolism

Animal group	Dose (mg/kg-BW)	No. of tested mice^①^	No. of dead^②^	Protection (%)	Mortality (%)
Control	-	24	23	4.2	95.8
WIB-801CE	200	20	15	25.0	75.0
	400	20	13	35.0	65.0
Aspirin	100	20	13	35.0	65.0

Protection (%) = (① - ②)/① × 100, Mortality (%) = ②/① × 100. No., number of tested mice; BW, body weight

### In vitro effects of WIB-801CE and cordycepin on fibrin clot retraction

**Fig. 6:**
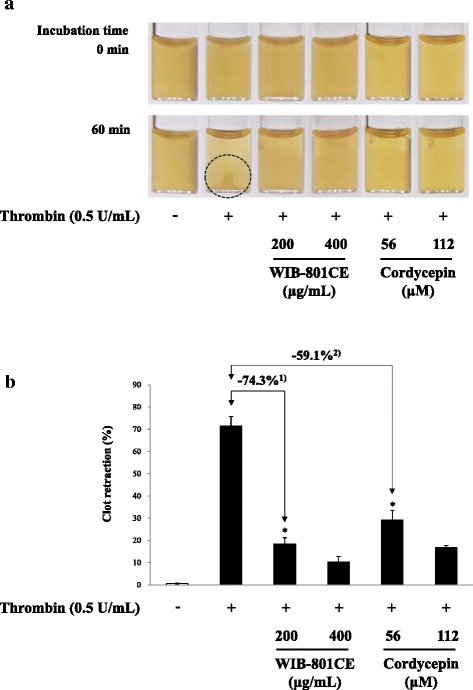
In vitro effects of WIB-801CE and cordycepin on fibrin clot retraction. **a** In vitro effects of WIB-801CE and cordycepin on thrombin-induced fibrin clot. **b** In vitro effects of WIB-801CE and cordycepin on thrombin-retracted clot retraction (%). Quantification of fibrin clot retraction was performed as described in “Methods” section. The data are expressed as the mean ± standard deviation (*n* = 4). ^*^ *p* < 0.05 versus the thrombin-stimulated platelets. ^1)^ Δ (%) = [(agonist + WIB-801CE 200 μg/mL) - agonist]/agonist × 100, ^2)^ Δ (%) = [(agonist + cordycepin 56 μM)- agonist]/agonist × 100

Fibrin clot retraction is a final index of platelet aggregation-mediated thrombotic formation, and is resulted from interaction of fibrin-platelet [24]. Thrombin stimulated the retraction of fibrin clot (Fig. 6a, dotted circle), but WIB-801CE (200, 400 μg/mL) inhibited it in a dose dependent manner (Fig. 6a and b). WIB-801CE (200 μg/mL) inhibited thrombin-induced fibrin clot retraction up to 74.3% (Fig. 6b). Cordycepin (56, 112 μM) corresponding to dose (200, 400 μg/mL) of WIB-801CE attenuated thrombin-induced fibrin clot retraction in a dose dependent manner (Fig. 6a and b). Cordycepin (56 μM) corresponding to 200 μg/mL of WIB-801CE inhibited thrombin-induced fibrin clot retraction (14.4 ± 5.1%) up to 59.1% (Fig. 6b).

### In vitro and ex vivo effects of WIB-801CE on NO production

**Fig. 7:**
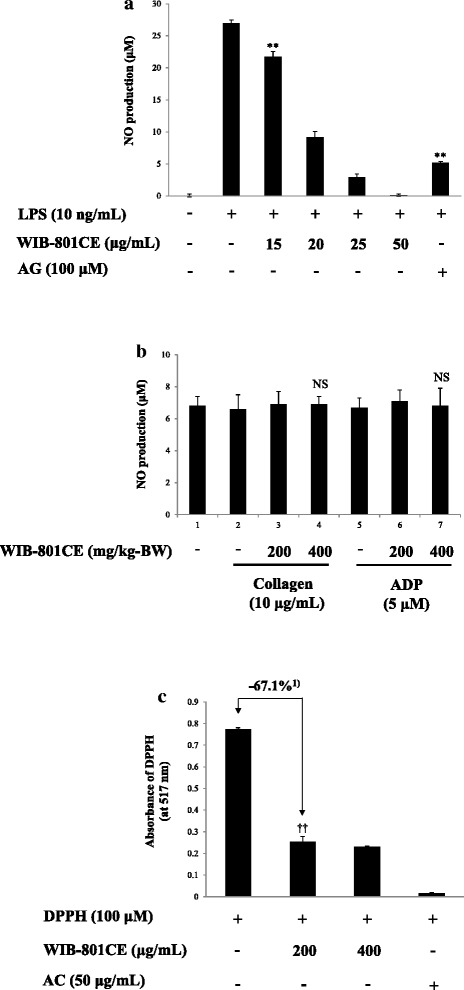
Effects of WIB-801CE on NO production, and antioxidation activity. **a** In vitro effects of WIB-801CE on LPS-induced NO production. **b** Ex vivo effects of WIB-801CE on collagen- and ADP-induced NO production. **c** In vitro effects of WIB-801CE on antioxidation activity. Measurements were carried out as described in “Methods” section. The data are expressed as the mean ± standard deviation (*n* = 4). ^**^ *p* < 0.001 versus LPS-stimulated RAW264.7 cells. ^††^ *p* < 0.001 versus the DPPH. NS, not significant versus the each agonist-stimulated platelets.^1)^ Δ (%) = [(DPPH + WIB-801CE 200 μg/mL) - DPPH]/DPPH × 100

It is well known that monocytes/macrophages and neutrophils produce various inflammatory mediators (i.e. NO, prostaglandin E_2_), and subsequently activate platelets to generate atherothrombosis [52, 53]. In recent, it is also reported that neutrophil-produced NO activates platelet in chronic renal failure [54]. Accordingly, we investigated whether WIB-801CE inhibits in vitro NO production in RAW264.7 macrophage cells. As shown in Fig. 7a, lipopolysaccharide (LPS), an activator of macrophages, potently produced NO as compared with that of normal. However, WIB-801CE dose (15 to 50 μg/mL)-dependently attenuated LPS-elevated NO production (Fig. 7a). iNOS inhibitor AG potently inhibited NO production (Fig. 7a).

The ex vivo NO levels in plasma from PRP of control (Fig. 7b, lane 2, 5) and WIB-801CE (200, 400 mg/kg-BW) stimulated with collagen (Fig. 7b, lane 3, 4) and ADP (Fig. 7b, lane 6, 7) were not changed as compared with that (Fig. 7b, lane 1) in plasma from control rat, in the absence of ADP, collagen and WIB-801CE. These mean that WIB-801CE (200, 400 mg/kg-BW) did not at least activate inflammatory iNOS in leukocytes (i.e. monocyte, macrophage, neutrophil) in vivo. Otherwise, the ex vivo NO levels would be increased in plasma from WIB-801CE (200, 400 mg/kg-BW) stimulated with collagen and ADP.

### In vitro effects of WIB-801CE on free radical scavenging activity

Reactive oxygen species (ROS) activate platelets and thrombogenesis [55–57]. In this study, we investigated whether WIB-801CE has an antioxidant effect scavenging ROS, which was evaluated as scavenging activity of free radical in DPPH, a stable free-radical molecules [25]. AC, a positive control, potently reduced the absorbance of DPPH. This means that AC has antioxidant effect by scavenging free radical in DPPH [25]. WIB-801CE also attenuated the absorbance of DPPH in a dose dependent manner (Fig. 7c). This result is indicated that WIB-801CE has antioxidant effect by scavenging free radical in DPPH like AC.

### Ex vivo effects of cordycepin on platelet aggregation

As shown in Table 5, When PRP (10^8^/mL) from control, was activated with ADP (10 μM), the aggregation rate was increased up to 75.7 ± 0.6%. But ADP-induced platelet aggregation significantly decreased by cordycepin (5, 10 mg/kg-BW) (Table 5). The inhibitory degree by 5 mg/kg-BW was 31.7%. This is higher than that (10.9%) by WIB-801CE (200 mg/kg-BW) (Fig. 2b).

**Table 5:** Effects of cordycepin administration in ADP-induced rat platelet aggregation

	Light transmission (%)	Inhibition (%)
Control + ADP (10 μM)	75.7 ± 0.6	-
Cordycepin (5 mg/kg-BW) + ADP (10 μM)	51.7 ± 0.6^**^	31.7
Cordycepin (10 mg/kg-BW) + ADP (10 μM)	47.3 ± 2.5^**^	37.4

The data are given as the mean ± standard deviation (*n* = 3). ^**^ *p* < 0.001 compared with control. Inhibition (%) = [(cordycepin + ADP) – control]/control × 100 (%)

## Discussion

WIB-801CE and its component cordycepin did not affect the cytotoxicity (determined as LDH leakage) and platelet activation (determined as platelet aggregation) to resting human platelets in vitro. These mean that there is no problem to evaluate the antiplatelet effects of WIB-801CE ex vivo. It is well established that various agonists (i.e. collagen, ADP, thrombin)-produced TXA_2_ generates circulatory disorder such as thrombosis, atherosclerosis, and myocardial infarction by stimulating platelet aggregation, vasoconstriction, and bronchoconstriction [5, 58, 59]. Therefore, it is essential to inhibit platelet aggregation and TXA_2_ production to prevent circulatory disorder in blood vessel. WIB-801CE attenuated ex vivo collagen- and ADP-induced platelet aggregation and TXA_2_ production.

These results were connected to the ex vivo inhibition of AA release and TXAS activity by WIB-801CE in collagen- and ADP-activated platelets. WIB-801CE did not also inhibit ex vivo collagen- and ADP-induced PLC_β3_ (Ser^537^, Ser^1105^), PLC_γ2_ (Tyr^1217^) phosphorylation. These mean that WIB-801CE would produce DG from phosphatidylinositol 4,5-bisphosphate in collagen- and ADP-activated platelets. DG is known to hydrolyze by p^38^ ^MAPK^-activated DG-lipase to release AA [45]. If so, it is considered that WIB-801CE may attenuate AA release by inhibiting p^38 MAPK^/DG-lipase pathway [45] without affecting inhibition of cPLA_2_ and PLC_β3_ [9, 10, 42, 43]. Because agonist-produced TXA_2_ enforces thrombus formation as a positive promoter [5, 58, 59], a compound or substance that inhibits the activity of COX-1 or TXAS, the production of TXA_2_ or the action of TXA_2_ is evaluated as antithrombotic agents. Many studies have been performed to discover therapeutic agents that can counteract the effects of TXA_2_. Various phytochemicals (i.e. epigallocatechin-3-gallate, caffeic acid, chlorogenic acid, caffedymine, sanguinarine) are known to inhibit COX-1 rather than TXAS to suppress the TXA_2_ production in vitro or ex vivo [60–65]. However, WIB-801CE inhibited the activity of TXAS rather than COX-1 ex vivo, which reflects that WIB-801CE inhibits the TXA_2_ production pathway from PGH_2_ rather than prostaglandin G_2_ production pathway from AA. In this study, we showed that WIB-801CE may involve in down-regulation of both p^38 MAPK^ phosphorylation to vanish the AA supply from DG and TXAS activity to block the TXA_2_ production from PGH_2_ ex vivo. Therefore, it is apparent that WIB-801CE can be beneficially used to prevent the TXA_2_-mediated thrombus formation in vivo.

It is well known that agonist-released serotonin stimulates irreversibly platelet aggregation, and subsequently causes the thrombosis as well as TXA_2_ [66–68]. WIB-801CE inhibited collagen- and ADP-induced serotonin release ex vivo, which reflects that WIB-801CE can inhibit the irreversible platelet aggregation in vivo. WIB-801CE potently inhibited ex vivo the phosphorylation of p^38^ ^MAPK^ and ERK2 (42 kDa), but not Ca^2+^-dependent myosin light chain (MLC) phosphorylation (Data not shown) that involves in serotonin release [14, 69–72] in collagen- and ADP-activated platelets. These results are allowed to consider that WIB-801CE seems to attenuate ex vivo serotonin release by inhibiting the phosphorylation of p^38^ ^MAPK^ and ERK2 rather than MLC in collagen- and ADP-activated platelets. This is similar to the reports that some phytochemicals (i.e. caffeic acid phenethyl ester, ginsenoside Rp1) inhibit collagen- and ADP-induced ATP release by phosphorylating p^38^ ^MAPK^ and ERK2 [39, 73].

In recent, we found that CE-WIB801C, n-butanol extracts from *Cordyceps militalis*, and cordycepin purified from CE-WIB801C has in vitro antiplatelet effect by inhibiting fibrinogen binding to glycoprotein IIb/IIIa *via* stimulation of cAMP-dependent phosphorylation of vasodilator-stimulated phosphoprotein (Ser^157^), and inhibition of phosphatidylinositol-3 kinase/Akt phosphorylation [74]. Antiplatelet effect of CE-WIB801C was involved in inhibition of collagen-induced serotonin release. Accordingly, we confirmed that the extracts from *Cordyceps militaris* have antiplatelet effect in vitro and in vivo.

The formation of fibrin clot by intrinsic and extrinsic blood coagulation factors together with platelet aggregation at injured blood vessels is another cause of thrombogenesis. WIB-801CE did not significantly prolong PT and APTT as compared with that by normal ex vivo. This reflects that WIB-801CE has no anticoagulant characteristics. WIB-801CE, however, weakly prolonged average PT as compared with that by normal. The prolongation of PT is associated with the reduction of coagulation factor VII production by inhibition of NADPH-vitamin K reductase [75]. The dose (1 mg/kg-BW) of warfarin, an inhibitor of NADPH-vitamin K reductase, that unlimitedly prologned PT is corresponded to high dose (60 mg/day) in case of giving to human (60 kg), which is more 12 fold than international normalized dose (5 mg/day) of warfarin [76]. In this study, because it is unknown whether the weak extension of average PT by WIB-801CE is clinically safety or risk, it is necessary to investigate PT using international normalized dose (5 mg/day) of warfarin, then the safety of WIB-801CE in the weak extension of PT should be evaluated in the future.

In addition, WIB-801CE without significantly affecting the prolongation of blood coagulation time may not influence on inhibition of fibrin production. If so, because the fibrin is retracted by the interaction with platelet aggregation [77], anybody may apprehend that the thrombus could be generated by WIB-801CE in vivo. But it is considered that its fear can be excluded because WIB-801CE inhibited both thrombogenic TXA_2_ production and serotonin release ex vivo, and thrombin-induced fibrin clot retraction in vitro. These results mean that WIB-801CE can strongly inhibit the fibrin clot retraction by down-regulating platelet activation without significantly affecting the blood coagulation. This is also evidenced as the effect that WIB-801CE inhibited collagen plus epinephrine-induced acute pulmonary thromboembolism in vivo, which is a marker of platelet aggregation-generated thrombogenesis. At the present study, however, it is unknown whether cordycepin in WIB-801CE contributed to the inhibition of acute pulmonary thromboembolism. These should be studied in the future.

As well as anticoagulants, antiplatelet drugs (i.e. aspirin, clopidogrel) also cause bleeding, and surprisingly may generate blood loss [21]. It is known that 20-40 mg/day of aspirin is clinically used in human to protect thrombotic disease [78]. The dose (100 mg/kg-BW) of aspirin, a positive control, seriously prolonged tail bleeding time of mice as compared with that by control. This aspirin dose (100 mg/kg-BW) is corresponded to high dose (6,000 mg/day) in case of giving to human (60 kg), which is more 300-150 fold than clinical dose (20-40 mg/day) of aspirin and impossible to compare with WIB-801CE in tail bleeding time of mice. At the present study, because it is unknown whether the significant extension of tail bleeding time by WIB-801CE is clinically safety or risk, it is necessary to investigate tail bleeding time using the clinical dose (20-40 mg/day) of aspirin as positive control, then the safety of WIB-801CE-prolonged tail bleeding time should be evaluated in the future.

Leukocyte-produced ROS oxidizes low density lipoprotein (LDL) in blood, then oxidized LDL (ox-LDL) is incorporated into macrophage to generate foam cell which damages vascular wall by inducing inflammation. Because platelet aggregation is caused at injured place of vascular wall, the inflammation by leukocyte-produced ROS and NO is the cause of thrombus formation. This means that the counteraction of agonists-induced platelet aggregation and leukocyte-induced inflammation might be contributed to the inhibition of thrombosis. WIB-801CE had ex vivo inhibitory effects on the release of serotonin that stimulates the uptake of ox-LDL into macrophage [79]. In addition, WIB-801CE inhibited NO production and elevated scavenging activity of free radical in DPPH in vitro. Therefore, it is anticipated that WIB-801CE would not activate inflammatory leukocytes in vivo as evidenced that WIB-801CE dose not affect ex vivo NO production. These results suggest that WIB-801CE might have antithrombotic effects by inhibiting inflammation *via* antioxidative action.

Because we could not identify cordycepin or its metabolites in PRP from WIB-801CE (200, 400 mg/kg-BW) rats, in the present study, we could not explain whether cordycepin in WIB-801CE was absorbed through intestine and subsequently involved in inhibition of platelet aggregation. This study should be performed in the future. Considering antiplatelet effects observed by blood collection of 2 h after final administration of WIB-801CE and 14 days after administration of cordycepin, although cordycepin is metabolized to an inactive 3'-deoxyhypoxanthinosine by adenosine deaminase in rat blood [80–82], it could be thought that unknown substances in WIB-801CE or cordycepin-derived unknown substance might involve in inhibition of platelet aggregation in an acute or chronic manner.

WIB-801CE (200, 400 mg/kg-BW)-dose independently exerted its inhibitory effects on platelet aggregation, TXA_2_ production, AA release, TXAS activity, serotonin release, p^38 MAPK^ phosphorylation and ERK2 phosphorylation in ADP-activated platelets. Considering the inhibition of ADP-induced platelet aggregation is a potential target to develop antithrombotic agent having antiplatelet characteristics [36. 37], it is thought that high dose of WIB-801CE (400 mg/kg-BW) might exert undesirable effect on platelets in vivo.

## Conclusion

Cordycepin-enriched WIB-801CE from *Cordyceps militaris* vanished ex vivo thrombogenic molecules (TXA_2_, serotonin), and their associated signaling molecules (AA, TXAS, p^38^ ^MAPK^, ERK2) in platelet aggregation. Furthermore, WIB-801CE inhibited in vivo acute pulmonary thromboembolism, an index of thrombotic formation, without having cytotoxicity and risk of serious bleeding, but with antioxidant and antiinflammatory activity. Therefore, we suggest that WIB-801CE may be a beneficial and effective substance to treat or protect thrombosis, atherosclerosis, and myocardial infarction *via* inhibition of platelet activation in vivo.

## Abbreviations

AA: Arachidonic acid
AC: Ascorbic acid
ADP: Adenosine diphosphate
AG: Amino guanidine
ANOVA: Analysis of variance
APTT: Activated partial thromboplastin time
ATCC: American type culture collection
BW: Body weight
COX-1: Cyclooxygenase-1
cPLA_2_: Cytosolic phospholipase A_2_
DG: Diacylglycerol
DPPH: 2,2-diphenyl-1-picrylhydrazyl
ECL: Enhanced chemiluminescence solution
EIA: Enzyme immunoassay
ELISA: Enzyme-linked immunosorbent assay
ERK: Extracellular signal–regulated kinase
HPLC: High performance liquid chromatography
HRP: Horseradish peroxidase conjugate
ICR: Institute of Cancer Research
IgG: Immunoglobulin G
iNOS: Inducible nitric oxide synthase
KRBC: Korean Red Cross Blood Center
LDH: Lactate dehydrogenase
LDL: Low density lipoprotein
LPS: Lipopolysaccharide
MAPK: Mitogen-activated protein kinase
MLC: Myosin light chain
MLCK: Myosin light chain kinase
NO: Nitric oxide
ox-LDL: Oxidized low density lipoprotein
PGH_2_: Prostaglandin H_2_
PLC _β3_: Phospholipase C_β3_
PLC _γ2_: Phospholipase C_γ2_
PPP: Platelet-poor plasma
PRP: Platelet-rich plasma
PT: Prothrombin time
PVDF: Polyvinylidene difluoride
ROS: Reactive oxygen species
SDS-PAGE: Sodium dodecyl sulfate-polyacrylamide gel electrophoresis
TXA_2_: Thromboxane A_2_
TXAS: TXA_2_ synthase
TXB_2_: Thromboxane B_2_
WIB-801CE: (*C*ompound from 20*08 First* Project of *B*ioteam, *W*han*i*n Pharm. Co., Ltd., Suwon, Korea), an ethanol extract from *Cordyceps militaris*-hypha
